# An insight into pyocyanin: production, characterization, and evaluation of its in vitro antibacterial, antifungal, antibiofilm and in vivo anti-schistosomal potency

**DOI:** 10.1186/s12866-025-04248-1

**Published:** 2025-08-23

**Authors:** Marwa M. Eltarahony, Salwa S. Younis, Sara A. Abdel Salam, Fadwa M. Arafa

**Affiliations:** 1 Environmental Biotechnology Department, Genetic Engineering and Biotechnology Research Institute, City of Scientific Research and Technological Applications (SRTA-City), New Borg El-Arab City, Alexandria 21934 Egypt; 2 Department of Medical Parasitology, Faculty of Medicine, Alexandria University, Alexandria, 21577 Egypt

**Keywords:** *Pseudomonas* sp., Antibacterial, Antifungal, Antiparasitic, MIC, Cytotoxicity, *Schistosoma mansoni*, Water-borne pathogens, Multidrug resistant (MDR)

## Abstract

**Background:**

Tackling the high demand for alternative therapeutic options, especially for multi-drug-resistant organisms, has awakened interest in naturally originated biomolecules such as pyocyanin (PYO). Herein, PYO-producing bacterium was isolated and PYO was extracted, characterized, and its antagonistic potency was scrutinized against a vast array of prokaryotic and eukaryotic water pathogens, either in free-living or biofilm lifestyles, both in vitro and in vivo.

**Results:**

Initially, *Pseudomonas* sp. MPF-2 that was screened from agricultural soil for PYO production, was characterized and identified. On King’s-A broth, the maximum productivity of PYO recorded 31.35 µg\ mL within 72 h. Subsequently, the extracted PYO exhibited maximum absorbance peaks at 277, 383 and 522 nm using UV–vis spectrophotometry. While FTIR spectrum accentuated the involvement of the main hallmark functional groups that characterize phenazine including O–H, C = N, aromatic C-H, C = C, CH3, C-O and C– O–C. Additionally, GC-MS analysis showed the major peaks of 1-hydroxyphenazine (hemi pyocyanin) and phenazine fractions at 206.1, 196.1 and 168.1 m/z. Upon employing it as an antibacterial agent, the crude PYO showed the lowest MIC (15.6 µg/mL) against *S. pneumoniae*, the highest MIC (500 µg/mL) versus *P. vulgaris*. While a considerable mycocidal potency was noticed against *C. albicans* and *C. tropicalis* at 125 and 62.5 µg/mL, respectively. As an antibiofilm agent, PYO displayed 98.11 ± 1.7, 88.04 ± 1.37 and 90.99 ± 2.13% inhibition for *B. cereus*, *P. vulgaris* and *C. albicans* biofilms, in that same order. Whereas, 61.65 ± 2.09, 40.86 ± 2.81 and 48.68 ± 2.56% eradication was detected at 1000 µg/mL for the pre-established biofilms of *B. cereus*, *P. vulgaris* and *C. albicans*, sequentially. Meanwhile, it didn’t show any significant toxicity regarding serum kidney and liver functions in orally treated mice at 25 mg/kg for 5 days. For antiparasitic potency, PYO-treated mice, at the juvenile stage, exhibited a significant reduction (*p* ≤ 0.05) in the total worm load (23.83 ± 2.79), with a reduction percentage of 34.41%, while PZQ-treated mice failed to show any significant reduction. Moreover, SEM micrographs emphasized the ultrastructural changes in *S. mansoni* juvenile and adult worms recovered from the PYO-treated mice. To the best of our knowledge, this is the first in vivo study to assess orally administered PYO as a therapeutic option against *S. mansoni.*

**Conclusion:**

The promising results encourage the recruiting of PYO in conquering the virulence of other pathogens in different environmental, medical and industrial applications.

## Background

Pathogenic microorganisms (e.g., bacteria, virus, fungi, parasites, etc.) are the main biological contaminants that can potentially jeopardize public health and the ecosystem, which subsequently devastate the economy. They are omnipresent on Earth and could spread easily via several routes, namely, contaminated water and food, infected persons, or nosocomial. They indwell the environment in both free-floating and biofilm lifestyles and could easily switch between them. However, their existence in sessile mode or biofilm architecture represents the most lethal stage. Thus, the utilization of antibiotics or synthetic drug therapy symbolizes the most commonly applied strategy to conquer such pathogenic executor’s threat. Despite their effectiveness with varied antagonistic mechanisms, the excessive and intensified usage of multiple drugs simultaneously in an uncontrolled manner would, in turn, generate the multidrug resistance (MDR) phenomenon [1]. However, the major obstacle of such phenomenon is its dissemination among different microbial groups in the environment via genetic mutations and horizontal gene transfer of tolerance genes [2]. Apart from the discovery of new compounds, science has also looked backwards through a new perspective, uncovering some bioactive compounds that were long abandoned but are now finding new uses. Intriguingly, natural bioactive compounds, such as bacterial pigments, have attracted scientists and biotechnologists than synthetic ones [1, 3, 4].

Pyocyanin (PYO) or blue pus is grouped among such naturally originating non-synthetic antimicrobial pigments, which gained momentum. PYO is mainly produced by different species of *Pseudomonas*, in particular *Pseudomonas aeruginosa (P. aeruginosa)*, and is considered a characteristic biomarker for its detection indirectly [5]. Further, it is the decisive, deadly toxin of *P. aeruginosa* that modulates its pathogenicity and virulence [6, 7]. PYO (C_13_H_10_N_2_O), a tricyclic extracellular secondary blue redox-active metabolite, affiliates to nitrogen-containing phenazine derivatives. It is produced during the late stationary stage, generating the most characteristic feature of blue-green diffusible pigment; shuttling the electrons and scavenging Fe (III) in iron-deficient milieu [8]. PYO exhibits a remarkable reactivity toward cellular reductants and interacts with signalling proteins, flavoenzymes, haem proteins, or transmembrane transporters [9]. As a result of these properties, PYO’s potential use in medicine was proven in both non-infectious diseases such as cancer as well as infectious diseases [10]. For instance, PYO exhibited strong and broad-spectrum antimicrobial properties against a wide array of bacterial pathogens such as methicillin-resistant *Staphylococcus aureus* (MRSA) and *Chlamydia* spp [11, 12], as well as fungi like *Candida albicans (C. albicans)* and *Cryptococcus* (species) spp [13, 14]. Additionally, it showed anti-plasmodial and anti-trichomonal effects that could expand these potential uses to more parasites [9, 15].

Accordingly, the current study focused on recruiting the biocide potency of PYO, as a naturally occurring biomolecule, in defeating a wide spectrum of infectious agents, including bacteria, fungi, and parasitic helminths. This was done by evaluating the in vitro antibacterial effect of PYO against a variety of Gram-negative bacteria (*Escherichia coli*, *Klebsiella pneumoniae*, *Proteus vulgaris*, and *Salmonella typhimurium*), and Gram-positive bacteria (*Bacillus cereus*, *Enterococcus faecalis*, *Staphylococcus aureus*, and *Streptococcus pneumoniae*) as well as its antifungal activity against *C. albicans* and *C. tropicalis.* Besides, its potentiality in thwarting biofilm development and its eradication strength against pre-formed biofilm were also assessed. Furthermore, the schistosomicidal activity of orally administered PYO was evaluated using parasitological and ultrastructural studies against the juvenile and adult stages of the Egyptian strain of *Schistosoma mansoni* (*S. mansoni*) in a murine model, which has not been studied before, to the best of our knowledge.

## Materials and methods

### PYO production, extraction and characterization

#### Screening of PYO pigment-producing isolate

As an environmental sample, about 1 gram of finely sieved agricultural soil was serially diluted by (0.85%) sterile saline solution, then 1 mL of dilutions were aseptically inoculated into King’s-A agar plates with the following ingredients (Himedia, India) (g/L): proteose peptone 20.0, MgCL_2_ 1.64, K_2_SO_4_ 10.0 and Agar 15.0 pH (7.3 ± 0.1). The inoculated plates were incubated at 30 °C for 24–48 h (Stuart-S1500, UK). After incubation, the plates were screened for bacterial colonies possessing blue-green pigmentation. The bacterial isolate that exhibited the highest PYO productivity was selected for further morphological characterization via scanning and transmission electron microscopy. The bacterial samples were prepared according to protocols described by Eltarahony et al., (2019) [16] and consequently, visualized by SEM (JEOL JEM-1230, Japan) and TEM (JEOL JSM 6360LA, Japan). Besides, biochemical properties were evaluated using Microbact-12E system (Thermo Scientific™, USA). While molecular identification was performed by 16S rRNA gene sequencing via an ABI 3730 automated sequencer (PerkinElmer/Applied Biosystems (Foster City, CA, USA). The generated sequence was submitted to Genebank to inquire about the similarity and obtain its accession number. For constructing a phylogenetic tree of the examined strain, MEGA- 6 software package was employed, and the neighbor-joining (NJ) approach was used with bootstrap analyses for 1000 replicates [17].

#### Production and extraction of PYO pigment

The enhanced productivity of PYO was performed by utilizing three types of media, namely, nutrient broth, King’s A and peptone water supplemented with glycerol. A 100 µl of bacterial lawn of cell density 3 × 10^8^ colony-forming unit/ml (CFU/ml) was inoculated to 150 ml of the previously mentioned media and incubated at 30 °C in an orbital shaker (150 revolutions per minute (rpm) for 72 h (Stuart-S1500, UK). A sample of 5 mL was drawn at a regular time interval (i.e., 24 h.) and analyzed for the maximum PYO production. For PYO extraction, the broth culture was collected and subjected to centrifugation at 11,000 × g for 15 min (Eppendorf-5810R, Germany). The clear supernatant was then transferred into a dark or fully wrapped bottle to prevent oxidation by light, mixed thoroughly with chloroform (1:2) (Thermo Scientific™, USA) and vortexed for 30 s (Eppendorf-5810R, Germany). The chloroform layer, which was blue-coloured, was separated and re-extracted by 0.1 N HCl (Thermo Scientific™, USA). The absorbance of the produced acidified pink upper layer was measured at 520 nm (Labomed model-double beam, LABOMED INC., USA). To determine the quantity of PYO, the optical density at 520 nm was multiplied by the extinction coefficient of 17.072. Such pink acidic fraction was thereafter neutralized with a minimum amount of 0.1 N NaOH, and re-extracted again by chloroform, followed by evaporation of this organic fraction. The obtained dried blue-colored PYO was dissolved in sterile distilled water and stored at 4 °C for further analytical and application stages [18–20].

### Characterization and structural analysis of PYO pigment

The chemical analysis of PYO solution was scrutinized through a number of characterization techniques as follows: [21, 22].

#### UV–vis spectrophotometry

The UV spectral measurement of the blue-colored crude PYO solution was analyzed, in quartz cuvette, using UV–vis spectrophotometer (Labomed model-double beam, LABOMED INC., USA) in a scanning wavelength range of 200–800 nm. Distilled water was used as a blank [23].

#### Fourier-Transform Infrared spectroscopy (FTIR)

The crude pyocyanin samples were encapsulated in 150 mg of KBr in the form of uniform disks and fixed in a sample holder. The spectra were measured using Shimadzu FTIR-8400 S, Japan, in the mid-IR region of 500–4500 cm^−1^ with a spatial resolution of 4 cm^−1^ [23].

#### Gas chromatography–mass spectrometry analysis (GC–MS)

The crude PYO was analyzed by Trace GC Ultra/MS ISQ Single Quadrupole (Thermo Fisher Scientific, USA). The sample was injected into the equipped TG-5MS fused silica capillary column with specifications of 30 m length, 0.25 mm ID and 0.1 mm film thickness. GC/MS detection was performed using an electron ionization system with ionization energy of 70 eV. Helium, as a carrier gas, was utilized at a constant flow rate of 1 ml/min. Besides, the injector and transfer temperature were programmed at 280 °C. However, the oven temperature was adjusted at 50 °C for 2 min, as an initial temperature, then uplifted to to150˚C for 2 min with an increasing rate of 7˚C/min, followed by increasing to 270 °C for 2 min with an increasing rate of 5˚C/min and ended by 310 ˚C for 10 min, as a final temperature, with an increasing rate of 3.5˚C/min. Finally, all the identified components were quantified using a percent relative peak area. The maximum peak was identified based on the comparison of mass-to-charge ratio characteristics with related organic compounds listed in the library data accompanying the software on the GC/MS system [24].

### In vitro applications of PYO pigment

#### Antimicrobial activity

The biocide activity of crude PYO was evaluated quantitatively by determining the minimum inhibitory concentration (MIC) and the minimum bactericidal concentration (MBC). Based on the Clinical and Laboratory Standards Institute (CLSI) 2018 standard approach, the microdilution assay in nutrient broth, for bacteria, and Sabouraud dextrose broth, for fungi, was employed. About 1 × 10^6^ CFU/mL of Gram-negative bacteria [*Escherichia coli* (ATCC 8739), *Klebsiella pneumoniae* (ATTC 700603), *Proteus vulgaris* (ATCC-8427), *Salmonella typhimurium* (ATCC 14028)], Gram-positive bacteria [*Bacillus cereus* (ATCC 33019), *Enterococcus faecalis* (ATCC 29212), *Staphylococcus aureus* (ATCC 29213)], *Streptococcus pneumoniae* (ATCC 6303) and yeast [*Candida albicans* (ATCC 10231) and *C. tropicalis* (ATCC 13803)] were inoculated in 96-well tissue culture plate in the presence of different pyocyanin concentrations (15.6–1000 µg/ml). In parallel, two controls were concurrently investigated: microbial culture lacking PYO as positive controls and culture media containing PYO and without any microbial culture as negative control. The inoculated microplates were incubated at 30 °C and 25 °C for 24 h and 48 h for bacteria and yeast, respectively. The filter-sterilised resazurin (0.015%, Sigma-Aldrich, USA) solution was mixed thoroughly with the content of each well after incubation and incubated for 2–4 h till the colour changed from purple to pink. The columns with blue/purple referred to the growth absence, whereas pink or colourless revealed the microbial growth [24]. The MIC value was recorded at the lowest concentration of PYO that prevented microbial growth (blue colour). However, MBC test was implemented by plating the suspension from each well of microtiter plates into nutrient agar and Sabouraud dextrose plates, followed by incubation at 30 °C and 25 °C for 24 h for bacteria and yeast, respectively. The lowest concentration with no visible growth on the agar plate was scored as MBC value [23].

#### Antibiofilm activity

As a standard assay, the microtitre plate method was utilized for detecting the biofilm development in the presence of PYO in different concentrations (31.25–1000 µg/ml). In brief, about 100 µL of *P. vulgaris* (ATCC-8427) (Gram-negative), *B. cereus* (ATCC 33019) (Gram-positive) and *C. albicans* (ATCC 10231) cultures were seeded in a sterile polystyrene 96-well microtiter plate. Besides, the control wells with microbial cultures devoid of PYO and also sterile media without microbial cultures were assessed as positive and negative controls, correspondingly. The plates were incubated for 24 h under static incubation at 30 °C. Upon complete incubation, the plates were emptied and washed twice with phosphate buffer saline (PBS) to remove free planktonic cells. Then, the plates were stained with 0.1% crystal violet (Sigma-Aldrich, USA) for 30 min at 30 °C, and the excess stain was eliminated by washing with PBS. Finally, the stained wells were solubilized by 95% ethanol, and the biofilm development/inhibition was assessed spectrophotometrically at 595 nm by a microplate reader (Sunrise™, TECAN, Switzerland). For determining biofilm inhibition, the formula of [Optical density (OD (control)-OD (test)/OD (control) × 100] was applied [25]. Additionally, the metabolic activity and the microbial viability of biofilm cells were also appraised using colorimetric MTT assay. Wherein, 150 µL of 0.25 mg/mL MTT solution (3-[4, 5-dimethylthiazol-2-yl]−2, 5-diphenyltetrazolium bromide, Sigma-Aldrich, USA) solution was mixed thoroughly with the overnight incubated and washed biofilm in each well after removing loosely attached cells. The microtiter plates were then incubated at 30 °C for 2–4 h; after incubation, the well content was removed, and 2% dimethyl sulfoxide (DMSO) was added for solubilizing insoluble purple formazan. The absorbance was detected at 570 nm by a microplate reader (Sunrise™, TECAN, Switzerland). The suppression of metabolic activity was expressed using the previously mentioned formula [26, 27].

#### Eradication of pre-established biofilm

The potency of PYO in different concentrations (15.6–1000 µg/ml) to disaggregate the already formed biofilms by the examined pathogens was assessed. Initially, about 200 µl of *P. vulgaris* (ATCC-8427) (Gram-negative), *B. cereus* (ATCC 33019) (Gram-positive) and *C. albicans* (ATCC 10231) cultures (~ 10^6^ CFU/ml) were transferred to a sterile 96-well microtiter plate and incubated for 48 h at 30 °C statically to allow the construction of biofilm. Then, the plate content was decanted aseptically to remove planktonic cells, and different concentrations of PYO were added to the 48 h-pre-established biofilms under aseptic conditions. Besides, positive and negative controls were run as mentioned previously in parallel. After treatment, the plates were incubated at 30 °C for 24 h, washed, stained, solubilized, measured spectrophotometrically, and the eradication percentage was calculated as mentioned formerly. The respiratory activity of the cells within the detached biofilm was also determined using MTT assay as mentioned in the previous section [27, 28].

### In vivo evaluation of anti-schistosomal activity

#### Animals

A total of 54 male Swiss albino mice, six to eight weeks old, weighing 20–25 g, were purchased from the animal house of the Medical Parasitology Department, Faculty of Medicine, Alexandria University, Egypt. These animals, used for safety assessment and experimental studies, were kept under standard light, temperature, and pathogen-free barrier conditions.

#### Drugs

PZQ ^®^ (SEDICO Pharmaceutical Company, 6th of October City, Egypt), 600 mg tablets, was purchased from the local pharmacy. Each tablet was weighed, ground, and dissolved in an aqueous suspension of phosphate-buffered saline (PBS), pH 7.4. It was given to mice by oral gavage in a dose of 200 mg/kg/day for 5 consecutive days [29]. PYO was biogenically produced in the laboratory of the Environmental Biotechnology Department, Genetic Engineering and Biotechnology Research Institute (GEBRI), City of Scientific Research and Technological Applications (SRTA-City), Alexandria, Egypt. A pilot study was conducted to determine the lowest effective oral dose of PYO, and 25 mg/kg/day for 5 consecutive days was selected.

#### Pre-study evaluation of PYO safety 

Twelve mice were divided equally into two groups as follows: Group 1, Normal non-treated mice; Group 2: Mice treated orally with 25 mg/kg of PYO for 5 days. Then, blood was collected from the jugular veins after the mice were anaesthetised by intraperitoneal injection of sodium pentobarbital at a dose of 40 mg/kg. After that, the unconscious mice were euthanized by cervical dislocation. Sera were used to measure kidney function biomarkers (urea and creatinine) as well as liver enzymes (alanine transaminase (ALT) and aspartate transaminase (AST) [30].

#### Animal grouping and experimental design

A total of 42 mice were randomly divided into three main groups as follows: Group I: 10 *S. mansoni*-infected non-treated mice; Group II: 16 infected PZQ-treated mice; and Group III: 16 infected PYO-treated mice. All mice were infected with 100 ± 10 cercariae per mouse, freshly shed from *Biomphalaria alexandrina snails*, using the paddling method [31, 32]. Sixteen mice in Groups II and III were further equally subdivided into 2 subgroups: the first one where drug administration was initiated on the 21 st day post infection (dpi) and continued for 5 days (against juveniles of *S.mansoni* life-cycle); and the second subgroup where drug administration was initiated on the 42nd dpi and continued for 5 days (corresponding to the adult stages of *S.mansoni*) [31]. Two infected mice from all subgroups were sacrificed 24 h following termination of the treatment schedule to collect the juvenile and adult worms for examination of ultrastructural morphological changes using scanning electron microscopy (SEM). Correspondingly, two infected non-treated mice from Group (I) were sacrificed on 21 st and 42nd dpi to recover juveniles and adult worms as control worms. While the remaining animals of all groups were sacrificed on 49th dpi. The therapeutic efficacy of the PYO in comparison with the corresponding infected non-treated control and PZQ-treated groups was assessed using parasitological and ultrastructural studies against experimental schistosomiasis mansoni in a murine model.

### Therapeutic activity against experimental *S. mansoni infection*

#### Parasitological study ( worm burden)

On the 49th dpi, each mouse was euthanized by intraperitoneal injection of 0.3 ml of pentobarbital/heparin solution (50 mL of 65 mg/mL sodium pentobarbital, 2.5 mL of 10, 000 U/mL heparin, and 75 mL water, Sigma-Aldrich, USA) [33] and subsequently the recovered adult worms from the hepatic and portomesenteric vessels of mice were counted in non-treated infected control (Group I) as well as treated infected groups (Group II and III) using a perfusion technique [34].

#### Ultrastructural study

Juvenile and adult worms were recovered from perfused infected mice 24 h after termination of the drug administration schedule. Obtained worms were fixed in cold 2.5% buffered glutaraldehyde phosphate, processed, examined under SEM and photographed [31].

### Statistical analysis

All the in vitro experiments were conducted in triplicate, and the results were expressed as mean ± standard error of mean (SEM). Tukey post-hoc analysis of variance (ANOVA) was employed to define the significance of treatments (*p* < 0.05) using Graphpad Instat software. As for the in vivo study, IBM SPSS software package version 20.0 was used to analyze the data that was fed into the computer (IBM Corp., Armonk, NY). The Shapiro-Wilk test was used to determine the normality of quantitative data then they were expressed as mean and standard deviation. For normally distributed quantitative variables, One way ANOVA test was utilized between the different studied groups, which was followed by Post Hoc test (Tukey) for pairwise comparison. Moreover, Student t-test was used for normally distributed quantitative variables to compare between two studied groups. The significance of the obtained results was judged at the 5% level. The percentage of reduction (% R) of mean adult and juvenile worm count was determined using the following equation:

$$\mathrm{Percentage}\;\mathrm{reduction}\;\left(\%\mathrm R\right)=\;\frac{\mathrm N\;-\;\mathrm n}{\mathrm N}\;\times\;100$$

Where (N) is the mean number of worms in the infected non-treated Group I and (n) is the mean number of worms in the infected treated group.

**Fig. 1:**
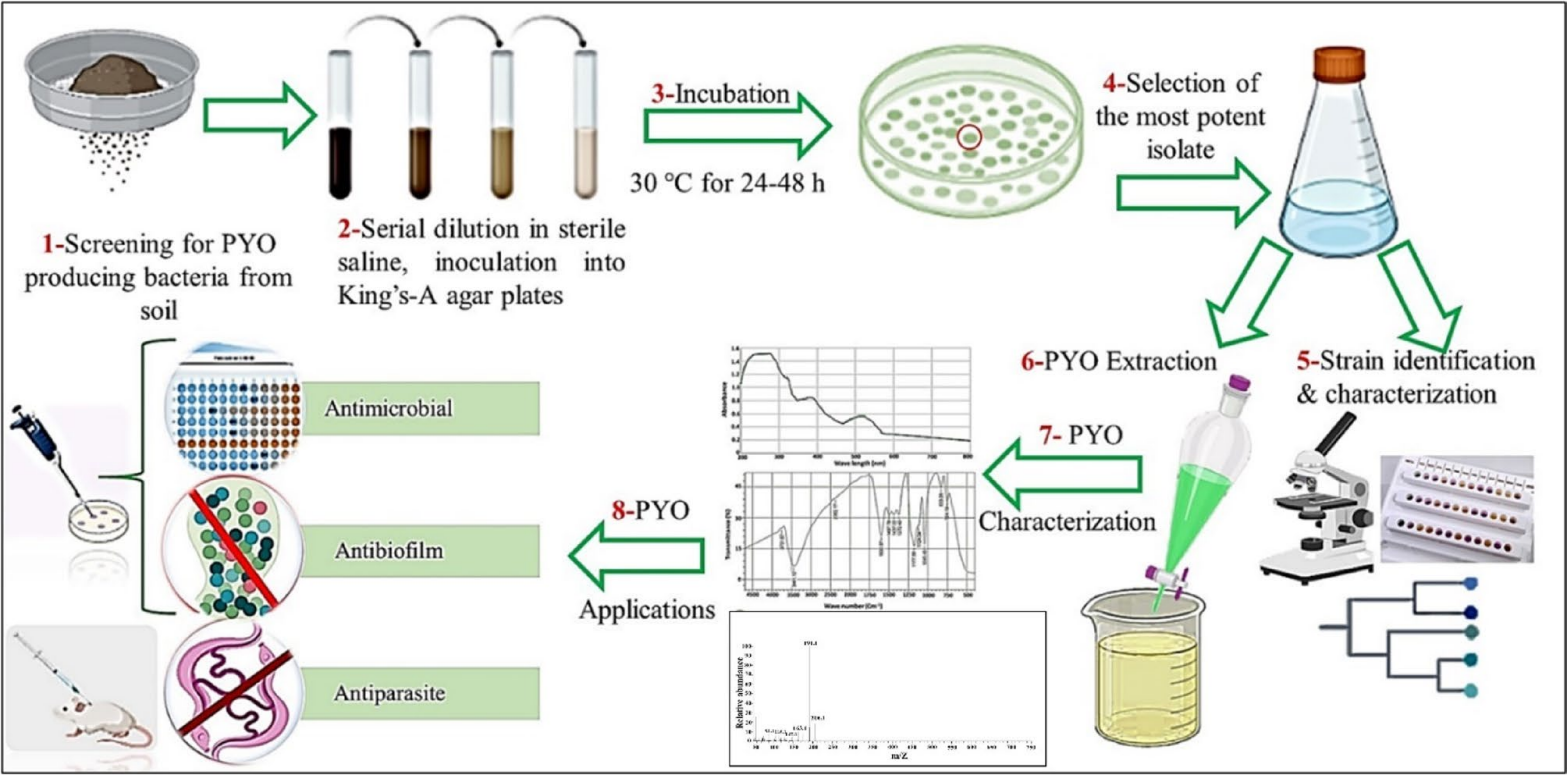
Schematic flow chart illustrating stages of the study beginning from the isolation of PYO-producing strain, passing through extraction and characterization, till application

In general, a schematic flow chart representing the experimental stages of the current study is depicted in Fig. 1.

## Results and discussion

### Screening and isolation of PYO pigment-producing isolate

**Fig. 2:**
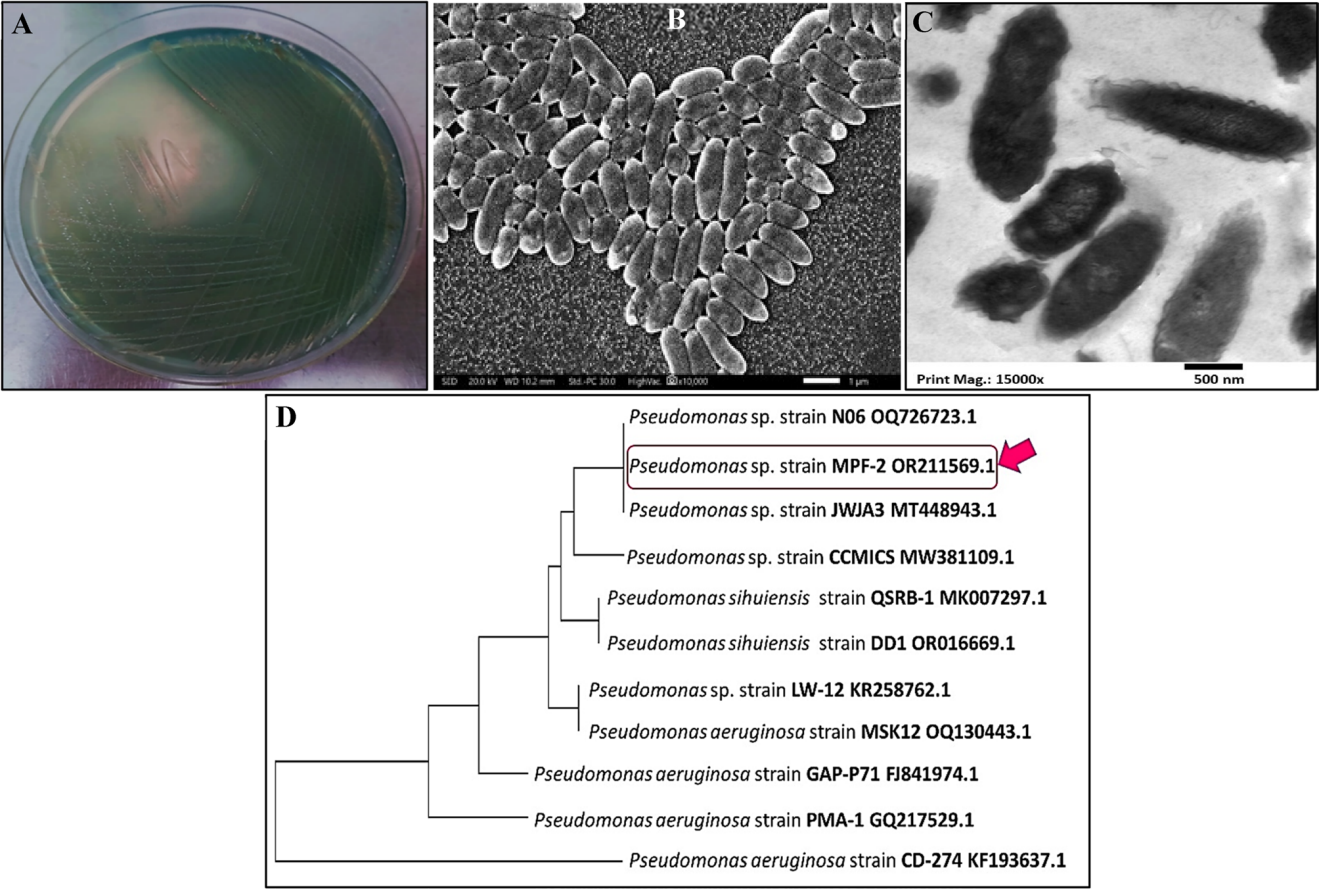
Morphological and cultural properties of PYO-producing *Pseudomonas* sp. MPF-2. **A** colony morphology on King’s A media, (**B**) SEM, (**C**)TEM and (**D**) Taxonomic relativeness using neighbor-joining phylogenetic tree of 16S rRNA gene

Remarkably, various phenazine compounds secreted by different microbial types displayed prominent antimicrobial and antiparasitic potency against a broad range of pathogens. From a biomedical perspective, PYO’s virulent nature and its capacity to cause oxidative stress in a variety of species make it a promising biological candidate. Despite this unending promise, the formulation of PYO-targeted therapies plus its utilization as a potent biotherapeutic molecule is still a subject of ongoing investigation [35]. Herein, a total of five isolates of pigmented bacteria were screened from the agricultural soil. Among them, the most potent isolate that produced a blue-green, fluorescent pigment was selected for further stages of the study. The purified isolate developed olive-color opaque flat round shape colonies with raised elevation and diffusible pigments on King’s-A agar plates (Fig. 2A), however, a distinctively fruity odor, particularly grape-like odor, was also detected. Besides, the electron microscopic investigation by SEM (Fig. 2B) and TEM (Fig. 2C) portrayed rod-shaped, non-spore forming and non-flagellated cells with cell dimensions assessed by 0.5–0.8 μm in width and 1–3 μm in length. The acquired 16S rRNA gene sequence was 1022 pb long, and the isolate was identified as *Pseudomonas sp.* MPF-2 and submitted to the National Center for Biotechnology Information (NCBI) GenBank under the accession number of OR211569. The examined strain exhibited 99% similarity with the nearest relatives of N06-OQ726723.1 and JWJA3-MT448943.1 which were screened from soil and petroleum-contaminated soil in China, respectively. Remarkably, as a taxonomic gold standard in defining the bacterial species’ phylogenies, the 16S rRNA sequence has long been employed. Broadly, the sequence of 16 S rRNA genes are varied among species of the bacterial domain, so such variation enables not only the discrimination among organisms at the genus level, through all major bacterial phyla, but also classifying strains to the species and subspecies levels [5]. Figure 2D symbolizes the phylogenetic tree, constructed by the Neighbour-joining (NJ) approach, of the inquired strain that matched other species of *Pseudomonas* such as *Pseudomonas aeruginosa* and *Pseudomonas sihuiensis.* On the other hand, the selected strain exhibited different biochemical traits. Wherein, it recorded positive results for mannitol fermentation, Simmon’s citrate utilization, o-nitrophenyl-ß-d-galactopyranoside hydrolysis and nitrate reduction. While tests of l-lysine decarboxylase, l-ornithine decarboxylase, H_2_S production, glucose fermentation, xylose fermentation, indol, uease, Voges-Proskaüer reaction and tryptophan deamination were negative.

### Production and structural analysis of PYO pigment

**Fig. 3:**
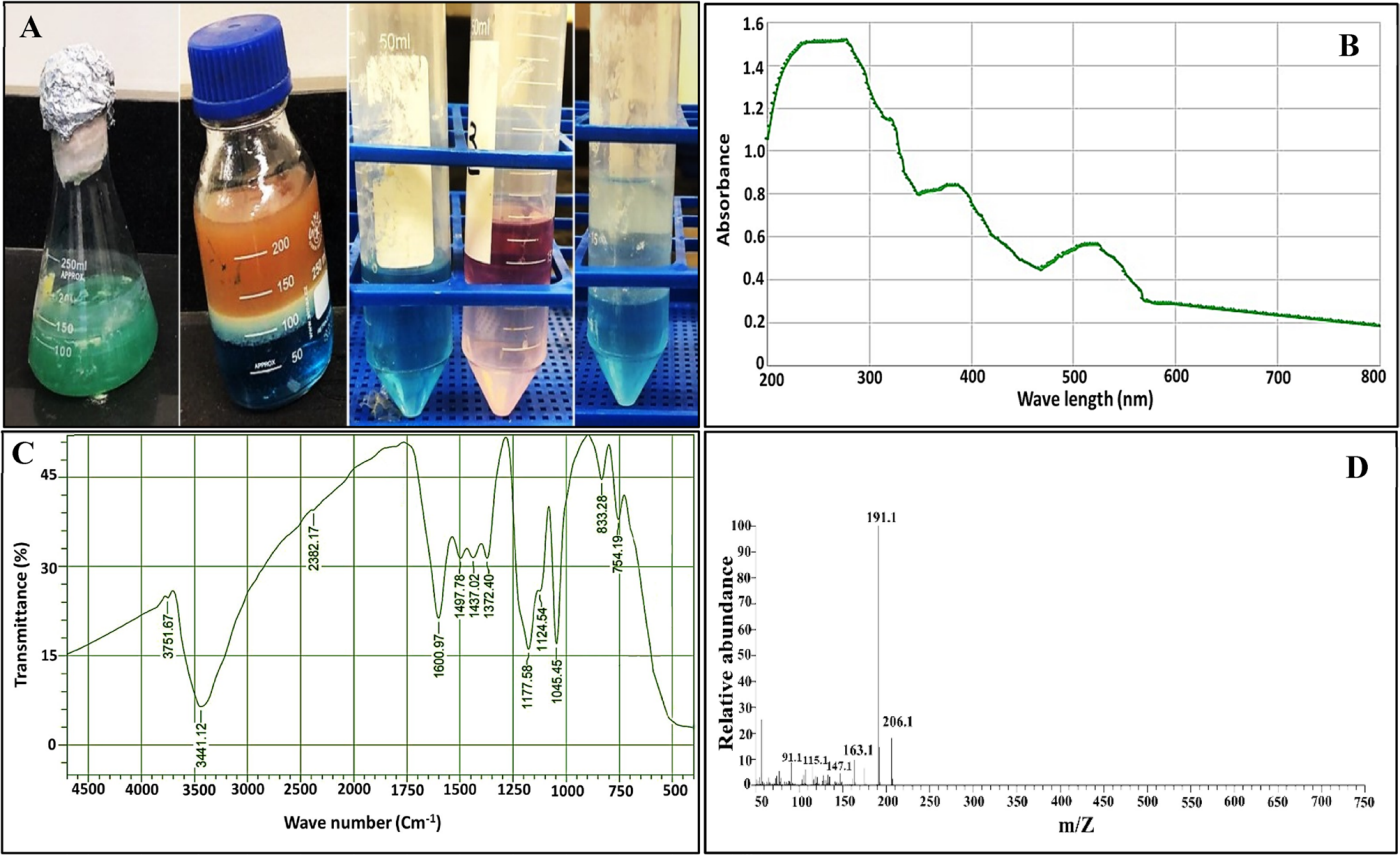
Extraction and structural properties of the extracted PYO from *Pseudomonas sp.* MPF-2. **A** Extraction stages, **B** -UV-spectrophotometer range, **C** FTIR pattern and **D** GC-MS analysis

The pigment production was implemented in King’s-A broth within 72 h of incubation, as it initiated after 18 h and increased gradually. Soluble PYO pigment was emphasized by a change in colour to bluish green in the presence of chloroform. Notably, the alteration in pigment color to deep pink was detected upon acidification of the chloroform mixture with HCl (Fig. 3A). Thereafter, the concentration of PYO was determined by measuring the optical density at 520 nm and multiplication by the extinction coefficient, which recorded 31.35 µg\ mL. Likewise, different studies reported that king’s broth is a better choice for maximum PYO production compared to other media such as nutrient broth, tryptone water and water peptone [36–38]. Nevertheless, the amendment of broth media with other supplements such as soya and sweet potato could enhance PYO productivity [17]. Strikingly, the obtained quantity of PYO in the current study is advantageous compared with other studies. Wherein, the PYO yield excreted by different species of *Pseudomonas* that were isolated from environmental and clinical samples was assessed by 0.1702 µg/ml [17], 9.3 µg/ml [39] and 10.42 µg/ml [18]. Additionally, the results revealed that 72 h incubation was the optimum time for PYO production, which is in harmony with those obtained by Barakat et al., 2015 [36].

As denoted by Koyun et al., 2022 [38], such heterogeneity in PYO yield could be ascribed to the nature of the screening sample, which is consequently resident of strains with different physiological and metabolic properties. Wherein, acyl-homoserine lactone (AHL) autoinducers that modulate and regulate the transcription and modification processes of PYO are highly influenced by environmental fluctuations in oxygen tension, pH, oxidative stress and temperature [39]. In this sense, Gahlout et al., 2021 [40] reported that *Pseudomonas sp.* JJTBVK (KF836502) excreted bluish-green PYO upon inducing the stress state through modifying the pH of the growth medium. On the other hand, it is important to mention that the production process of PYO could be described as time-dependent. Wherein, the production of this pigment began after 18 h of incubation, which uplifted gradually during 72 h. However, the prolonged incubation period, namely, more than 72 h, adversely affected PYO production by our strain. Actually, *Pseudomonas* sp. possess the ability to produce other pigments at longer incubation duration, such as pyorubin (red- brown) and pyomelanin (light brown), which could hinder the accuracy in the extraction step and calculate its concentration inaccurately. This result agreed with that obtained by Darwesh et al., 2019 [37] and Saleem et al., 2021 [41].

Interestingly, PYO symbolizes with its redox properties and solubility dualism, which facilitates its extraction. Wherein, PYO fluctuates its colour from blue in neutral and basic pH to reddish pink in acidic pH. Under alkaline conditions, it is present in its oxidized form, so it is highly soluble in organic solvents such as chloroform. On the other hand, higher solubility is recorded in aqueous phase in acidic conditions. That could be attributed to its basic feature of one of the nitrogen atoms, which undergoes protonation as referred to by Mudaliar et al., 2024 [35]. In general, such duality in solubility pH changing makes the water/chloroform extraction process accurate, successful and highly effective [41, 42].

The physicochemical traits of the crude PYO were scrutinized; the absorbance spectrum of PYO was monitored in the spectral range of 200–800 nm using UV/Vis spectrophotometer. As observed in Fig. 3B, the maximum absorbance peak was detected at 277 nm, however, other two peaks were also noticed at 383 and 522 nm, which are considered primarily indicative of PYO presence. As declared by Thukkaram et al., 2024 [43], the peaks at 278 nm and 400 nm could be attributed to π– π* and n– π* transition, respectively. Our results agreed with those obtained by Marey et al., 2024 [23] who also reported that the characteristic peak of standard PYO exists at 382 nm. In the same context, several studies documented that PYO dissolved in 0.1 N HCl exhibited a number of peaks at 204, 242, 277, 387.5, and 521.5 nm [37, 39]. Whereas the UV spectrum of PYO dissolved in methanol was displayed at 316, 367 and 700 nm [37, 39]. While six maximum absorption peaks were detected for PYO dissolved in chloroform solvent at 285, 310, 320, 325, 495 and 700 nm as revealed by Darwesh et al., 2019 [37]. Meanwhile, Koyun et al., 2022 [38] found that the maximum absorption peak of it was detected at 300 and 370 nm for water and 95% ethanol, respectively. Collectively, the leverage of pH generates colour change of pigment upon using different solvents such as HCl, methanol, ethanol and chloroform, etc., which ultimately alters absorption maximum peaks in the examined range of UV–vis spectrum [38, 43].

Nonetheless, Watson et al., 1986 [44] recorded the same UV–vis spectrum profile of PYO irrespective of the utilized solvents. Broadly, as manifested by Verma et al., 2015 [45], the most characteristic absorption spectra of PYO displayed two unique maxima peaks in the UV range, while at least one in the visible range; attributing the differences in the exact position of absorption peaks to the variation in the position and nature of substituents on the heterocyclic ring.

On the other hand, FTIR analysis was employed to characterize the functional groups of PYO. Figure 3C represents several bands in the spectrum of 500–4500 cm^−1^, wherein the peaks at 3751 and 3441 cm^−1^ could be ascribed to stretching vibrations of O–H groups [46]. Besides, the vibration band at 2382 cm^−1^ indicated the presence of C = N stretching [40], while Mullaiselvan et al., 2020 [5] and Hamad et al., 2020 [47] attributed such peak to C-H aromatic bond of PYO molecule.

Meanwhile, the detection of a spectral peak at 1600 cm^−1^ could indicate the presence of an alkenyl C = C stretch [17, 40, 46–48]. However, the wavenumber of 1497 and 1437 cm^−1^ corresponded to benzene ring’s C = C stretch as revealed by Gahlout et al. 2021 [40]. Remarkably, the coexistence of the C-H bond of the alkyl group (methyl) could be implied from the peak at 1472 cm^−1^ [38]. Whereas, the absorbance peaks centered at 1177 and 1124 cm^−1^ could be assigned to C-O stretching [48]. Further, the identified band at 1045 cm^−1^ could be ascribed to the stretching vibration of C– O–C group as referred by Arafa et al., 2023 [49]. Other spectral peaks were detected at 833 and 745 cm^−1^, which are related to aromatic–CH in the molecule as highlighted by Prabhu et al., 2014 [8] and Hamad et al., 2020 [47]. Generally, the obtained FTIR profile of the examined PYO distinctively characterized the aromatic hydrocarbon ring (i.e., phenazine). Remarkably, the obtained FTIR pattern of the current study was congruent with previous scholars [23, 38, 47]. Strikingly, our FTIR profile was devoid of the toxic cyano (C ≡ N) and acetylenic (C ≡ C) groups, which were expressed in the range of 2000–2250 cm^−1^; implying the safety of the pigment, in particular for biological activity [22]. Meanwhile, it possessed the essential functional groups that are responsible for binding actively with different biomolecules to fulfil its role successfully, as would be displayed in the subsequent applications section.

Furthermore, the mass spectrum analysis of the crude PYO emphasized the presence of PYO compound in the examined sample. Wherein, distinct molecular ion peaks at 206.1, 191.1 and 163.1 m/z were detected in the spectrum along with other minor peaks that weren’t taken in consideration owing to their negligible amounts (Fig. 3D). The intense peak at 206 m/z could be ascribed to 1-hydroxyphenazine (hemi pyocyanin). Varnava et al., 2024 [50] unveiled that the peaks at 196.1 and 168.1 m/z could be assigned to 1-hydroxyphenazine (hemi pyocyanin) and phenazine fractions, respectively. Intriguingly, their simultaneous presence in the GC-MS spectrum could be explained by the conversion of 1-hydroxy-N-methyl phenazine to 1-hydroxyphenazine via the loss of a methyl radical (•CH3). Generally, the obtained result closely approximates the overall PYO spectrum profile referred to by Thukkaram et al., 2024 [43], and agrees with that found by Qasim, 2019 [51], who detected an intense molecular ion peak at 205 m/z with chemical formula of C_12_H_8_N_2_O that was attributed to hemi pyocyanin. On the other hand, Gahlout et al., 2021 [40] and Marey et al., 2024 [23] demonstrated that the purified PYO possess a protonated molecular ion at 210 m/z that defines the molecular weight of 210 KDa.

### In vitro applications of PYO pigment

#### Antimicrobial activity

Herein, resazurin blue dye accompanied broth microdilution assay to determine the minimum dosage that possesses the ability to cease microbial growth. Such applied approach mainly relies on the microbial metabolism of oxidoreductases in reducing resazurin into resorufin and altering its colour from blue to pink [24]. Despite each tested strain responding differently to the PYO, the Gram-positive bacteria seemed more susceptible than the Gram-negative group, as demonstrated in Table 1. Wherein, MIC values against the examined pathogens, in their free-living or planktonic phase, ranged from 15.6 to 500 µg/mL. The lowest MIC dosage of crude PYO (15.6 µg/mL) was shown against *S. pneumoniae*; however, the highest MIC value (i.e., 500 µg/mL) was detected against *P. vulgaris*, inferring its highest resistance capability. Broadly, the highest antimicrobial potency of PYO was noticed against Gram-positive bacteria compared to other examined strains of Gram-negative bacteria. Meanwhile, the lowest concentration of PYO that ceased the microbial growth over an extended period of incubation, namely MBC, was also elucidated in Table 1. Both MIC and MBC values were concurred, except for *K. pneumoniae*,* S. pneumoniae* and *C. tropicalis*. Notably, no microbial growth was observed in the dosage of 1000 µg/mL.

**Table 1:** Antimicrobial activity of PYO against human pathogens expressed as MIC and MBC

Class	Examined pathogen	MIC (µg/ml)	MBC (µg/ml)
Gram-negative bacteria	*Escherichia coli* (ATCC 8739)	250	250
	*Klebsiella pneumoniae* (ATTC 700603)	125	250
	*Proteus vulgaris* (ATCC-8427)	500	500
	*Salmonella typhimurium* (ATCC 14028)	125	125
Gram-positive bacteria	*Bacillus cereus* (ATCC 33019)	62.5	62.5
	*Enterococcus faecalis* (ATCC 29212)	62.5	62.5
	*Staphylococcus aureus* (ATCC 29213)	31.25	31.25
	*Streptococcus pneumoniae* (ATCC 6303)	15.6	31.25
Yeast	*Candida albicans* (ATCC 10231)	125	125
	*C. tropicalis* (ATCC 13803)	62.5	125

The obtained data revealed the efficacy of PYO extracted from *Pseudomonas sp.* MPF-2 (OR211569), which could serve as a broad-spectrum antibiotic, antagonizing pathogens in a dose-dependent modality, by recording MIC and MBC values in the range of 15.6 to 500 µg/mL. Wherein, de Oliveira et al., 2021 [52] referred that higher antimicrobial potency of any biocide agent was considered at 50 > MIC < 500 µg/mL, while the moderate and weak activity were considered at 500 > MIC < 1500 and MIC > 1500 µg/mL, correspondingly. Additionally, as a general observation, Gram-positive bacteria seemed to be more vulnerable to the PYO than Gram-negative bacteria, which was aligned with numerous previous investigations. Those results could be ascribed to the differences in the cell wall structure between both groups, in particular their lipid content, which eventually generates a variation in PYO sensitivity pattern (i.e., weak, intermediate and potent). As denoted by Marey et al., 2024 [23] the lipid content of peptidoglycan and lipopolysaccharide layers of Gram-negative cell wall is higher than that of Gram-positive, rendering them higher resistant. Besides, surface charge and electronegativity of the cell wall could also be taken into account. Wherein, the lower negatively charged peptidoglycan molecules of Gram-positive cell walls give most PYO constructions more affinity for the positive ions they produce, thereby elevating the absorption and total ion buildup, which ultimately causes intracellular harm [6]. On the other hand, the higher productivity levels of catalase and superoxide dismutase enzymes by both bacterial groups may also contribute to their susceptibility/tolerance profile [41]. Nevertheless, the antagonistic potentiality of PYO against Gram-negative bacteria could be exerted through inactivating their efflux pumps that extrude drugs [42].

In comparison, a study conducted by Shouman et al., 2023 [39] found that MIC values of PYO against *E. coli*, *P. mirabilis* and *K. pneumoniae* ranged from 150 to 300 µg/ml, while for MRSA strains, MIC values ranged from 40 to 70 µg/ml. In addition, Ashour et al., 2021 [53] manifested that PYO extracted from *P. aeruginosa* AS6 significantly impeded the bacterial growth in the range of MIC (6–9 mg/mL), and MBC values ranged from 10 to 18 mg/mL. In the same sense, Gahlout et al., 2021 [40] indicated that MIC values for suppressing the growth of *S. aureus* and *K. pneumonia* were 400 and 1000 µg/ml, respectively.

Remarkably, an eminent mycogenic effectiveness was represented by PYO of *Pseudomonas sp.* MPF-2 against the examined species of *Candida*, which were categorized among white fungi associated with COVID-19 post-infections. In this regard, Ashour et al., 2021 [53] mentioned that the range of 9–18 and 18–36 mg/mL of PYO represented MIC and MFC ranges, correspondingly, that inhibited the growth of *C. albicans and C. gelberta*. Besides, Shouman et al., 2023 [39] declared the antifungal potential of PYO that was exerted by 250–300 µg/ml against clinical isolates of *C. albicans*, reflecting the superior performance of PYO in the current study. Interestingly, the “red death” phenomenon of *C. albicans* by the action of *Pseudomonas sp.* is tightly relevant to the antifungal traits of PYO as referred by Shouman et al., 2023 [39]. Additionally, Thukkaram et al., 2024 [43] mentioned that MIC value in the range of 32–64 µg/mL is sufficient to inhibit the fungal growth of *Candida species* and *Cryptococcus neoformans*. Broadly, the variations of results reported by different scholars could be attributed to the differences in the physiology, metabolic performance and resistance profile of the examined pathogens. Nonetheless, the environmental conditions, such as pH and ionic strength, also possess a substantial effect on properties of the bacterial surface and their electrical charge, which vary not only intraspecies but also interspecies, as highlighted by de Oliveira et al., 2021 [52].

Generally, PYO exerts its antagonistic activity mainly based on its advantageous low molecular weight (210 Da zwitterion), which enables it to penetrate easily the cell membrane, causing its disruption by the dint of its redox potential, like all phenazine compounds [5, 35, 40, 48]. Wherein, as a redox-active biomolecule, it modulates redox cycles by arresting the normal transport of electrons in the respiratory chain, interacting with nicotinamide adenine dinucleotide hydrogen (NADH) and nicotinamide adenine dinucleotide phosphate hydrogen (NADPH) converting it to its reduced form; subsequently, inducing oxidative stress and instigating elevated levels of the intracellular oxygen-free radicals (ROS) such as superoxide (O_2_^−^) and hydrogen peroxide (H_2_O_2_). Thereafter, the deleterious cytotoxic effects of ROS lead to cell death in a programmed multistep complex mechanism, including specific destruction of DNA, lipid peroxidation, NAD(P)H depletion, enzymatic inhibition, suppression of the ion’s interaction with the membrane, respiration blocking and oxidative damage to cell cycle components that are responsible for metabolic activity, reproduction and their regulation [35, 42, 48]. Incontestably, the producing strains of PYO could resist its detrimental influence through continuous yielding of superoxide dismutase and catalase [54].

#### Antibiofilm activity

**Fig. 4:**
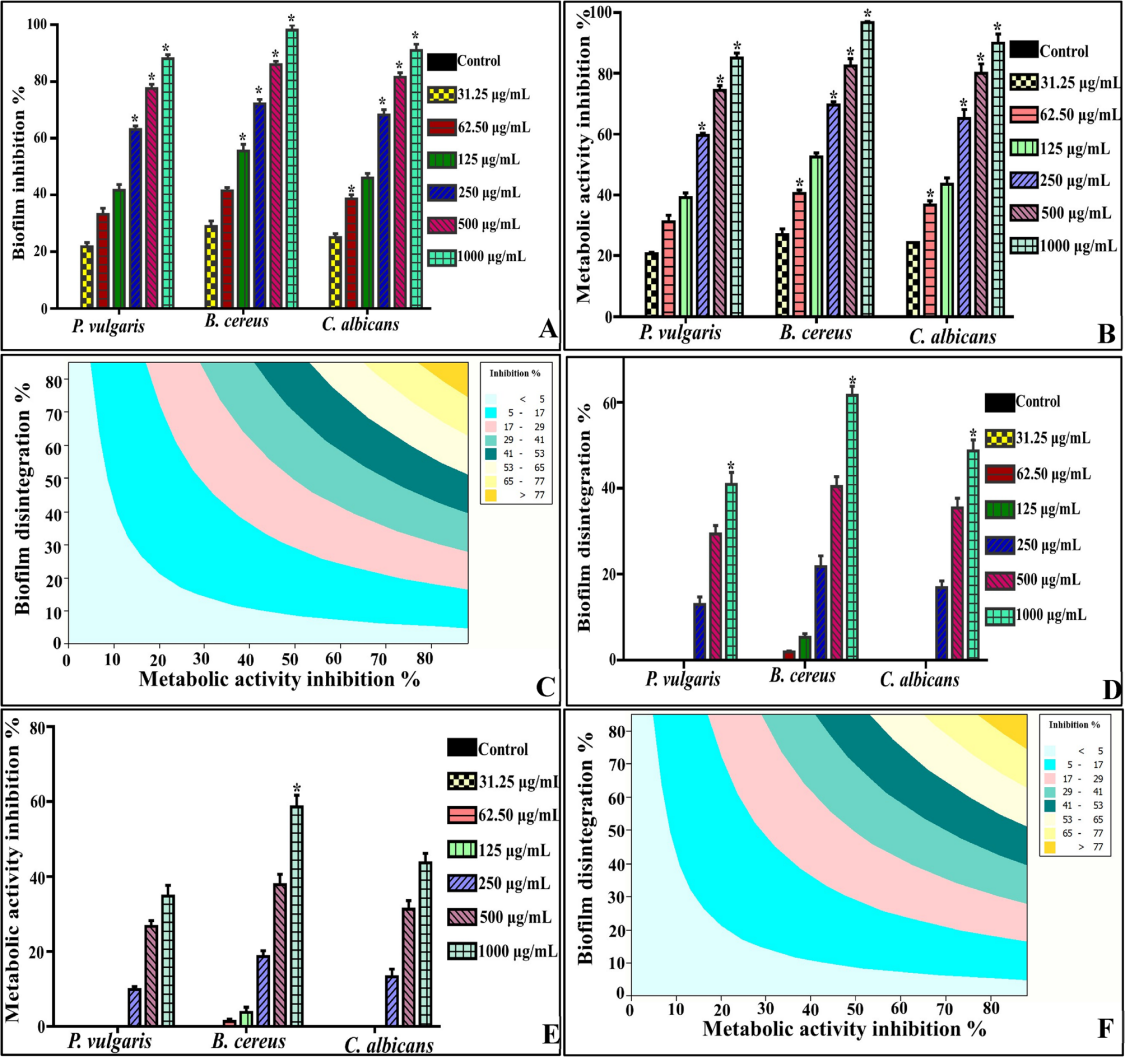
Antibiofilm activity and eradication potency of PYO in different concentrations against biofilm–forming pathogens. **A** Biofilm development inhibition, **B** Metabolic activity of biofilm cells inhibition, **C** Contour plot showing the correlation between biofilm inhibition and metabolic activity inhibition during biofilm growth, **D** Eradication of pre-formed biofilm, **E** Metabolic activity of pre-formed biofilm cells inhibition and **F** Contour plot showing the correlation between eradication of pre-formed biofilm and metabolic activity inhibition

Clearly, the biofilm proliferation, in all tested pathogens, was enfeebled relative to the control, in a microbe/concentration-dependent modality of inhibition (Fig. 4A). In coincidence with the planktonic mode of life, the biofilm of Gram-positive pathogen, namely, *B. cereus*, was the most pathogen affected adversely by all examined concentrations of PYO. Wherein, its biofilm synthesis was insignificantly suppressed at the low concentrations of PYO (i.e., 31.25 and 62.5) by percentages of 28.9 ± 1.86 and 41.5 ± 1.05, respectively. On the other hand, a moderate inhibition pattern was detected in the biofilm growth of *P. vulgaris* and *C. albicans* at the exact concentrations in the range of 21.7 ± 1.5–41.6 ± 2.04% and 25 ± 1.38–46 ± 1.57% in the same order, reflecting their superior tolerance. However, a significant (*P* ≤ 0.05) and more evident blockage in biofilm growth was exerted by 1000 µg/mL of PYO, as symbolized by ANOVA, against *B. cereus*, *P. vulgaris* and *C. albicans* by 98.11 ± 1.7, 88.04 ± 1.37 and 90.99 ± 2.13%, sequentially. Besides, the metabolic activity profile of the active cells enclosed in the biofilm structure was illustrated in Fig. 4B. Where PYO suppressed the cellular activity of *B. cereus* cells more than *P. vulgaris* and *C. albicans* in a linear concentration-dependent behavior. As detected, the highest significant inhibition (*P* ≤ 0.05) was recorded at 1000 µg/mL of PYO by 96.7 ± 0.32, 85.0 ± 1.62 and 89.9 ± 2.93% for *B. cereus*,* P. vulgaris* and *C. albicans*, respectively. Hereby, the metabolic activity of all examined cells within biofilm matrix were significantly and intuitively correlated with biofilm biomass (*r* ≥ 0.99, *P* = 0.00), reflecting thwarting leverage of PYO, in different applied dosage, on active cells (Fig. 4C). Sensibly, the correlation between the results of biofilms and their metabolic activities unveiled the importance of C.V. and MTT assays in studying biofilm. Wherein, C.V. assay sheds light on the slimy biofilm biomass that encompasses polysaccharides with negatively charged molecules, which are evenly distributed on the surface of live and dead cells as well. In parallel, MTT assay gives insinuation into the active cells that are still live, whatever their spatial existence in the multilayer architecture of biofilm. Stickling, El-Naggar et al., 2023 [55] pointed out that the cells at the top layer of biofilm exhibited higher metabolic performance than those enclosed in the inner layers.

In comparison, Shouman et al., 2023 [39] reported that 150–300 µg/mL of PYO deterred the formation and growth of biofilm developed by a number of food-borne and human pathogens (e.g., *E. coli*,* K. pneumoniae*,* K. oxytoca*,* P. mirabilis*,* S. aureus*,* C. albicans*, etc.) in the range of 61 to 83%. Likewise, Saleem et al., 2021 [41] found a similar antibiofilm profile of PYO for suppressing the formation of biofilm by 81, 78 and 76% for *B. cereus*, *P. aeruginonsa* and *K. pneumoniae*; recommending that the inactivation of viable bacterial cells is associated with reduction of biofilm biomass.

#### Eradication of pre-established biofilm

The disintegrating profile of already-established biofilm by the action of PYO in different concentrations (31.25–1000 µg/mL) showed notoriously tolerance to almost all treatment dosages. Wherein, the lower concentrations ranged from 31.25 to 125 µg/mL displayed very limited or failed completely in destroying the structure of pre-formed biofilms for all examined pathogens (Fig. 4D). While more pronounced and significant eradication effect was detected upon uplifting the applied dosage till reached to the maximum detachment percentage at 1000 µg/mL, which assessed by 61.65 ± 2.09, 40.86 ± 2.81 and 48.68 ± 2.56% for *B. cereus*, *P. vulgaris* and *C. albicans*, correspondingly. Meanwhile, the viability of active cells associated with pre-formed biofilm exhibited similar performance (Fig. 4E). Notably, the most substantial inhibition percentage (*P* ≤ 0.05) was assessed by 58.64 ± 3.09, 34.86 ± 2.81 and 43.68 ± 2.56%, respectively, at 1000 µg/mL. Similarly, a significant correlation was noticed between the cell viability and disruption of biofilm matrix upon elevating the PYO dosages (*r* ≥ 0.99, *P* = 0.00) (Fig. 4F). Such curtailing in eradication potency considered being intuitive, could be attributed to the intricacies derived from the adequate interaction and contact between PYO and microbial cells that are tightly bound together in a durable and low permeable EPS lattice. However, the stationary cells in the dormant zone of biofilms are characterized by their higher resistance, which augments the antimicrobial dosage required for their defeat, as reflected by Eltarahony et al., 2021 [56]. Thus, higher inhibitory dosages were entailed for destroying the already-formed biofilm compared to the developed counterparts. The planktonic cells at early stages of growth and colonization seem to be more vulnerable than dormant cells that are encapsulated inside a polymeric barrier. In agreement with our results, Saleem et al., 2021 [41] reported that 50 µg/ml of the extracted PYO showed significant damping of the biofilm formation against *B. cereus S. aureus* and *P. aeruginonsa* by 81, 80 and 78%, respectively. Whereas the exact dosage disrupted their pre-formed biofilms by 77, 76 and 74%, respectively. Conversely, de Oliveira et al., 2021 [52] documented that 37.5 µg/mL of PYO endorsed a greater adherence for *S. aureus* by 43%; unveiling the selective pressure role of PYO against microbial cell, which responded quickly by the formation biofilm and prompt adhesion in the shortest time, hence, the adherence stage during the establishment of biofilm is very critical, especially under stressful conditions.

Out of these results, the strategy followed by PYO in exerting its antibiofilm activity could be inferred. It could cause lysis for the initial planktonic stage, destructing sessile/aggregated and maturation phases, disrupting extracellular polymeric substances (EPS) matrix, weakening EPS hydrophobicity, damaging cell-to-cell adhesion/interaction, inhibiting cell-to-surface adhesion, modifying the biotic/abiotic surface properties and devastating quorum sensing system [41]. Intriguingly, the recent medications undergo continuous and advanced development of anti-virulence strategies, which are based on enhancing the efficacy of quorum-sensing blockers. Wherein, quorum sensing system (i.e., cell-to-cell communication strategy) represents the main virulence factor for most biofilm-forming pathogens, thereby, an effective pathway for conquering microbial infection with a little risk of resistance development. As manifested by Abdelaziz et al., 2023 [42], PYO displayed unique anti-quorum sensing activity.

### In vivo evaluation of anti-schistosomal activity

#### Pre-study evaluation of PYO safety

Prior to any in vivo experimentation, safety must be initially established. Wherein, the tested compound should not impact these healthy cells to be even considered a viable chemotherapeutic option [35]. In the current study, mice treated orally with 25 mg/kg of PYO for 5 days (Group 2) showed no statistically significant alteration of neither serum kidney function biomarkers (urea and creatinine) nor liver enzymes (alanine transaminase (ALT) and aspartate transaminase (AST)) in comparison to normal non-treated control (Group 1) (Table 2). Likewise, Laxmi and Bhat, 2016 [57] indicated that PYO was not cytotoxic against normal cell line, and even at high concentrations, the viability of around 80% indicated it can be safely utilized in food intended for human consumption. Furthermore, Ashour et al., 2021 [53] suggested that PYO powder can be introduced as a natural food additive to boost broiler chicken’s growth and combat pathogenic bacteria without any adverse effects on their productivity or meat quality. The previous evidence augments the results of our initial safety study, which showed normal kidney and liver functions for the evaluated dose and duration.

**Table 2:** Serum kidney function biomarkers (urea and creatinine) and liver enzymes (ALT and AST) in non- infected PYO- treated group in comparison to normal non-treated control group

Group	Group 1	Group 2	t	*p*
Biomarker				
**Urea (mg/dl)**				
Mean ± SD	15.83 ± 2.93	15.17 ± 3.19	0.377	0.714
Min.– Max.	12–19	11–20		
**Creatinine (mg/dl)**				
Mean ± SD	0.96 ± 0.15	1.03 ± 0.12	0.848	0.416
Min.– Max.	0.79–1.18	0.83–1.2		
**ALT (IU/L)**				
Mean ± SD	27.67 ± 2.80	28 ± 3.41	0.185	0.857
Min.– Max.	24–31	24–32		
**AST (IU/L)**				
Mean ± SD	24.67 ± 3.61	25 ± 4.47	0.142	0.890
Min.– Max.	22–31	20–31		

Group 1: Normal non-treated group

Group 2: Non-infected PYO-treated

t: Student t-test

p: *p* value for comparing between the studied groups

*Statistically significant at *p* ≤ 0.05

#### Parasitological study (Worm burden)

Table 3 shows *S. mansoni* adult worms collected on the 49th dpi from infected groups (I, II and III), which were counted under the dissecting microscope. As regards the total worms collected from mice treated on 21 st dpi (at the juvenile stage), PYO treatment showed a statistically significant superiority over PZQ at this stage. PYO-treated mice exhibited a statistically significant reduction in the mean total worm load (23.83 ± 2.79), with a reduction percentage of 34.41%, in comparison to the non-treated control (Group I) (*p* ≤ 0.05), while PZQ-treated mice failed to show any statistically significant reduction of total worm burden.

Results of worm burden from mice treated on the 42nd dpi revealed the reversal of the situation, with PZQ treatment taking the upper hand over PYO at the adult stage. The percentage of reduction of the total worm load was 94.96% (mean ± SD = 1.83 ± 0.75) in PZQ-treated mice while it reached 49.08% (mean ± SD = 18.50 ± 3.45) in PYO-treated mice, nonetheless, they both showed a statistically significant reduction in comparison to the non-treated control (Group I) (*p* ≤ 0.05).

**Table 3:** *Schistosoma mansoni* worm burden in infected mice treated with PZQ and PYO in comparison with infected non-treated control against the juvenile and adult stages of infection

	Group I	Group II	Group III	F	*p*
**Juvenile**					
Mean ± SD	36.33 ± 3.98	35.83 ± 4.26	23.83 ± 2.79	21.567^*^	< 0.001^*^
Min.– Max.	29–40	30–41	20–27		
% R		1.38	34.41		
p _0_		0.971	< 0.001^*^		
p _1_		< 0.001^*^			
**Adult**					
Mean ± SD	36.33 ± 3.98	1.83 ± 0.75	18.50 ± 3.45	189.112^*^	< 0.001^*^
Min.– Max.	29–40	1–3	14–24		
% R		94.96	49.08		
p _0_		< 0.001^*^	< 0.001^*^		
p _1_		< 0.001^*^			

Group I: Infected non-treated control

Group II: Infected PZQ-treated

Group III: Infected PYO-treated

Percentage of reduction (% R)

F: F for One way ANOVA test, Pairwise comparison between each 2 groups was done using Post Hoc Test (Tukey)

p: *p* value for comparing between the studied groups

p_0_: *p* value for comparing Group I and each other group

p_1_: *p* value for comparing Group II and Group III

*Statistically significant at *p* ≤ 0.05

#### Ultrastructural study

**Fig. 5:**
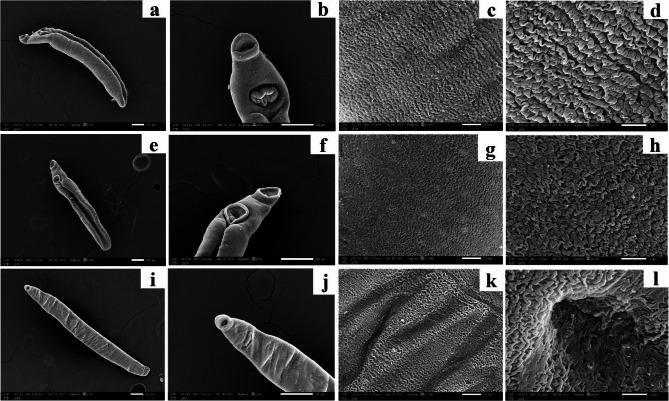
Scanning electron micrographs of *S. mansoni* juvenile worms recovered from infected mice: (**a**-**d**) normal juvenile worm revealed (**a**) ventral gynaecophoric groove (x100); (**b**) normal oval oral and ventral suckers (x250); (**c** & **d**) dorsal tegumental surface showing deep folds with indentations (x1500 and x4000, respectively); (**e**-**h**) juveniles retrieved from PZQ-treated mice revealing (**e**) preserved normal body features (x100); (**f**) normal suckers’ morphology and size (x250); (**g** & **h**) apparently normal integumental surface (x1500 and x4000, respectively); (**i**-**l**) juvenile worms recovered from the PYO-treated mice demonstrating (**i**) elongated furrowed stiff body with an evident failure of the development of gynaecophoric groove (x100); (**j**) an edematous oral sucker and invaginated ventral sucker (x250); (**k**) marked deep transverse tegumental furrows and creases (x1500); (**l**) numerous focal deep crater-like holes (x4000)

The SEM images of juvenile worms recovered from infected non-treated mice (Group I) revealed a ventral gynaecophoric groove (Fig. 5a) as well as oval oral and ventral suckers (Fig. 5b). The dorsal tegumental surface showed deep folds with indentations (Fig. 5c and d). Comparable morphological changes were observed in juveniles retrieved from PZQ-treated mice (Group II). They preserved their normal body features (Fig. 5e), suckers’ morphology and size (Fig. 5f) as well as the integumental surface (Fig. 5g and h). On the other hand, profound ultrastructural changes were detected in juvenile worms recovered from the PYO-treated mice (Group III) showing elongated furrowed stiff body with an evident failure of the development of gynaecophoric groove (Fig. 5i) as well as an edematous oral sucker and invaginated ventral sucker (Fig. 5j). The tegument exhibited marked deep transverse furrows and creases (Fig. 5k) and numerous focal deep crater-like holes (Fig. 5l).

**Fig. 6:**
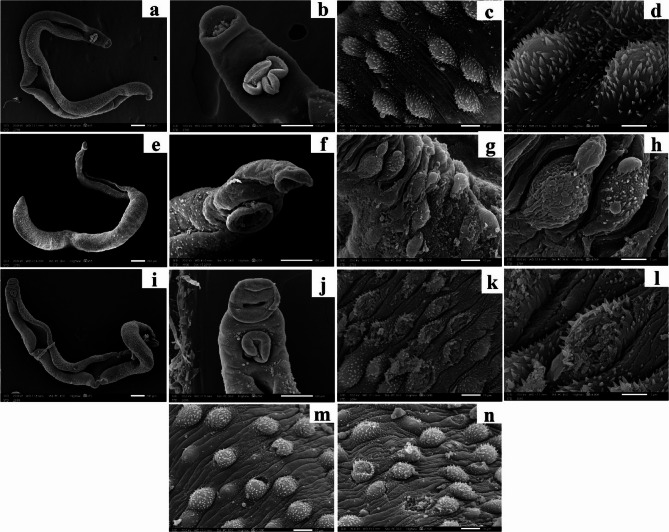
Scanning electron micrographs of *S. mansoni* adult worms recovered from infected mice: (**a**-**d**) adult male worm recovered from infected non-treated mice demonstrating (**a**) normal ventrally folded gynecophoric canal (x55); (**b**) well-developed oral and acetabular suckers (x250); (**c** & **d**) the mid-dorsolateral tegumental surface showing numerous evenly distributed tubercles and inter-tubercular ridges with sharp apically directed spines (x1500 and x4000, respectively); (**e**-**h**) PZQ-treated adult male worm revealing: (**e**) a twisted body and a constriction near its posterior end (x55); (**f**) normal oral and acetabular suckers (x250); (**g**) dorsal tegumental peeling with appearance of subtegumental tissue and partial loss of spines (x1500); (**h**) disorganized destructed tubercles with focal surface blebbing (x4000); (**i**-**n**) PYO-treated adult male worm exhibiting (**i**) elongated deformed body, widened gynecophoric canal with complete loss of its tone and twisted posterior end (x55); (**j**) prominent reduction in acetabular sucker’s size relative to the edematous oral sucker (x250); (**k** & **l**) integument appeared extensively disrupted with short blunt disorganized spines (x1500 and x4000, respectively); (**m** & **n**) sloughed, cracked and pinched off tubercles exposing the subtegumental tissues (x1500)

On the other hand, adult male worms recovered from infected non-treated mice (Group I) exhibited ventrally folded gynecophoric canal (Fig. 6a) as well as well-developed oral and acetabular suckers (Fig. 6b). The mid-dorsolateral tegumental surface showed numerous evenly distributed tubercles and inter-tubercular ridges with sharp apically directed spines (Fig. 6c and d). Micrographs of PZQ-treated adult male worms (Group II) revealed a twisted body with a constriction near its posterior end (Fig. 6e) and normal suckers (Fig. 6f). Dorsal tegumental peeling with the appearance of subtegumental tissue and partial loss of spines (Fig. 6g) as well as disorganized destructed tubercles with focal surface blebbing were observed (Fig. 6h). Finally, marked ultrastructural alterations were observed in PYO-treated adult male worms (Group III). The elongated deformed body showed widening of the gynecophoric canal with complete loss of its tone, twisted posterior end (Fig. 6i), and prominent reduction in acetabular sucker’s size relative to the edematous oral sucker were observed (Fig. 6j). The integument appeared extensively disrupted with short blunt disorganized spines (Fig. 6k and l). Some tubercles were sloughed and cracked, while others were pinched off exposing the subtegumental tissues (Fig. 6m and n).

Schistosomiasis, a chronic debilitating disease caused by the genus *Schistosoma*, is considered one of the neglected tropical diseases (NTDs), with at least 251.4 million people requiring preventive chemotherapy in 2021 [58]. Its transmission could be executed through dermal contact with urinary or fecally contaminated freshwater containing the intermediate host snails [59]. Remarkably, research suggested that schistosomiasis is much more prevalent than formerly assumed, which was brought to light using novel and more sensitive diagnostic techniques [60, 61]. Although World health Organization (WHO) has previously projected the year 2025 for the global eradication of schistosomiasis [62], this goal is currently unattainable as control measures were hindered by the COVID-19 pandemic, and the work to alleviate its impacts influenced NTD interventions, especially treatment coverage for schistosomiasis [58]. Since the 1970 s, praziquantel (PZQ) has been the cornerstone in schistosomiasis therapy; however, it is not effective against the intra-mammalian juvenile stages, and because of its widespread use and mass drug administration (MDA), several reports of PZQ resistance in endemic areas have emerged [63].

Although the anti-schistosomal activity of PYO has been previously explored [64], it was reported to be effective in combination with PZQ and by the parenteral route of administration rather than the oral route, which is an obvious limitation, and only against the adult stage. This encouraged the authors to proceed to animal experimentation via the oral route and against the juvenile and adult stages. In the current study, there was an evident reduction of total worm burden in both juvenile and adult PYO-treated subgroups. This reduction was specifically remarkable as it surpassed the reference drug PZQ in the juvenile stage, which is significant since PZQ is known to be non-efficacious against immature worms. This can be justified by the fact that PYO-induced reactive oxygen species (ROS) production resulted in oxidative stress, which has a number of detrimental effects, such as a drop in NADPH levels, inhibition of crucial enzymes, DNA damage, interference with membrane potential, and ultimately oxidative damage to several elements involved in cell cycle regulation [42]. This eventually led to ultrastructural deformities such as tubercular sloughing and destruction, exposing the subtegumental tissues, as witnessed by SEM, which accelerated parasite death and elimination by the immune cells. It is well-known that the onset and course of parasitic infections are significantly influenced by oxidative stress in both the parasite struggling to survive and the host that is being attacked. The ROS, primarily superoxide anion (O_2_ •−) and hydrogen peroxide (H_2_O_2_), are utilized by the host in huge quantities to combat the developing infection. However, in order to survive in the host, the parasite builds defence mechanisms to counteract ROS [65]. In a continuous effort to tip the scales in favour of the host’s immune system to facilitate parasite elimination, researchers have been investigating compounds that enhance oxidative stress within the parasites, taking advantage of their compromised antioxidant defences for developing more potent and targeted antiparasitic treatments [65]. As a naturally originated compound that is known for its oxidant effect, PYO offers a better alternative than synthetic drugs.

For helminths to interact and react to their external environment, fast-synaptic transmission in their neuromusculature is essential. Inhibition of this neuromuscular activity can cause loss of muscle function and interference with attachment, feeding, and mating, among other vital functions that are necessary for parasite growth and maturation, resulting in parasite death [66]. Another proposed mechanism of action for PYO is its ability to counteract the activities of cellular acetylcholinesterase (AChE) [35]. This anti-AChE characteristic is ascribed to its distinct redox chemistry, small size, and zwitterionic nature, which enable it to cross eukaryotic cell membranes. PYO’s action is reportedly equivalent to that of AChE inhibitors in use today [38]. In the cholinergic system of flatworms, acetylcholine (ACh) induces muscular contraction via membrane depolarization, by signalling through cation-selective nicotinic acetylcholine receptors (nAChRs), due to an influx of Na^+^, K^+^ or Ca^++^. The principal function of AChE is to stop transmission at cholinergic synapses by quickly hydrolyzing the neurotransmitter ACh into choline and acetate, thus allowing ions to move along electrochemical gradients in or out of cells, in that manner, AChE plays a significant role in modulating the interaction between ACh and the parasite receptors (nAChRs). When AChE activity is inhibited, the high levels of ACh cause continuous stimulation of nAChR, leading to its desensitization and closing of ion channels, which may produce evident consequences such as flaccid paralysis [67–69]. In *Schistosoma*, this enzyme is found in the muscle and on the tegument membrane of blood-dwelling adults as well as schistosomula [66]. In the present work, inhibition of the synaptic transmission in the neuromusculature of adult worms, leading to paralysis, might explain the observed loss of tone and widening of the gynaecophoric canal. It may also justify the apparent oedema and effacement of suckers which interferes with parasite attachment and feeding leading to eventual death.

## Conclusion

Recently, the misuse of antimicrobial agents has led to the prevalence of multi-drug resistance phenomenon. In particular, after the emergence of COVID-19 pandemic, intensive concerns regarding secondary infections have been raised. Therefore, seeking other natural alternatives, especially biocidal pigments, is highly urgent. PYO was extracted from terrestrial *Pseudomonas* sp. MPF-2 and characterized by UV–vis spectrophotometry, FTIR and GC-MS. All such techniques emphasized the presence of hallmarks that characterize phenazine, in which PYO is affiliated. Thereafter, the antagonistic properties of PYO were accentuated against a wide array of Gram-positive, Gram-negative and fungal pathogens via MIC and MBC assays. Additionally, PYO displayed a considerable antibiofilm activity and also biofilm eradication potency against *B. cereus*, *P. vulgaris* and *C. albicans*. Furthermore, as antiparasitic agent, for the first time, PYO was orally administered to Swiss albino mice and exhibited a successful potency against not only the adult stage but also the juvenile stage of *S. mansoni* with a reduction percentage reaching to 34.41% and with an acceptable margin of safety, comparing to reference drug PZQ; thereby paving the way for its utilization as a feasible chemotherapeutic agent. On this basis, due to the multifaceted antagonistic nature of PYO, future studies utilizing “Synergistic therapy” by hybridizing PYO with antibiotics or nanomaterials are highly recommended for combating water-borne pathogens. That in turn would boost the biocide potential, relative to powerless traditional treatments, with a low chance of developing resistance phenomena.

## Data Availability

All data generated or analyzed during this study are included in this published article. The 16 S-rRNA sequencing technique was performed and the data were deposited in the GenBank database under the accession number of OR211569 (https://www.ncbi.nlm.nih.gov/nuccore/OR211569.1/).

## Abbreviations

ACh: Acetylcholine
AChE: Acetylcholinesterase
AHL: Acyl-homoserine lactone
ALT: Alanine transaminase
ANOVA: Analysis of variance
AST: Aspartate transaminase
CFU: Colony-forming unit
CLSI: Clinical and Laboratory Standards Institute
DMSO: Dimethyl sulfoxide
dpi: Day post infection
ELISA: Enzyme-linked immunosorbent assay
EPS: Extracellular polymeric substances
FTIR: Fourier-Transform Infrared spectroscopy
GC-MS: Gas chromatography–mass spectrometry
GEBRI: Genetic Engineering and Biotechnology Research Institute
H_2_O_2_: Hydrogen peroxide
MBC: Minimum bactericidal concentration
MDA: Mass drug administration
MDR: Multi-drug resistance
MIC: Minimum inhibitory concentration
MRSA: Methicillin-resistant Staphylococcus aureus
MTT: 3-[4,5-dimethylthiazol-2-yl]-2,5-diphenyltetrazolium bromide
nAChRs: Nicotinic acetylcholine receptors
NADH: Nicotinamide adenine dinucleotide hydrogen
NADPH: Nicotinamide adenine dinucleotide phosphate hydrogen
NCBI: National Center for Biotechnology Information
NJ: Neighbor-joining
NTDs: Neglected tropical diseases
O_2_^−^: Superoxide
OD: Optical density
PBS: Phosphate buffer saline
PYO: Pyocyanin
PZQ: Praziquantel
ROS: Oxygen-free radicals
rpm: Revolutions per minute
SEM: Scanning electron microscopy
spp: Species
SRTA-City: City of Scientific Research and Technological Applications
TEM: Transmission electron microscopy
WHO: World health Organization
